# The use of master protocols for efficient trial design to evaluate radiotherapy interventions: a systematic review

**DOI:** 10.1093/jnci/djae084

**Published:** 2024-04-27

**Authors:** Alexandra Gilbert, Robert Samuel, Daniel Cagney, David Sebag-Montefiore, Julia Brown, Sarah R Brown

**Affiliations:** Leeds Institute for Medical Research, University of Leeds, St James’s University Hospital, Leeds, UK; Leeds Cancer Research UK Clinical Trials Unit, Leeds Institute of Clinical Trials Research, University of Leeds, Leeds, UK; Leeds Institute for Medical Research, University of Leeds, St James’s University Hospital, Leeds, UK; Radiation Oncology, Mater Private Hospital, Dublin, Ireland; Royal College of Surgeons in Ireland, University of Medicine and Health Sciences, Dublin, Ireland; Leeds Institute for Medical Research, University of Leeds, St James’s University Hospital, Leeds, UK; Leeds Cancer Research UK Clinical Trials Unit, Leeds Institute of Clinical Trials Research, University of Leeds, Leeds, UK; Leeds Cancer Research UK Clinical Trials Unit, Leeds Institute of Clinical Trials Research, University of Leeds, Leeds, UK; Leeds Cancer Research UK Clinical Trials Unit, Leeds Institute of Clinical Trials Research, University of Leeds, Leeds, UK

## Abstract

The aim of this review was to highlight why the use of master protocols trial design is particularly useful for radiotherapy intervention trials where complex setup pathways (including quality assurance, user training, and integrating multiple modalities of treatment) may hinder clinical advances.

We carried out a systematic review according to Preferred Reporting Items for Systematic reviews and Meta-Analyses (PRISMA) guidelines, reviewing the findings using a landscape analysis. Results were summarized descriptively, reporting on trial characteristics highlighting the benefits, limitations, and challenges of developing and implementing radiotherapy master protocols, with three case studies selected to explore these issues in more detail.

Twelve studies were suitable for inclusion (4 platform trials, 3 umbrella trials, and 5 basket trials), evaluating a mix of solid tumor sites in both curative and palliative settings. The interventions were categorized into 1) novel agent and radiotherapy combinations; 2) radiotherapy dose personalization; and 3) device evaluation, with a case study provided for each intervention. Benefits of master protocol trials for radiotherapy intervention include protocol efficiency for implementation of novel radiotherapy techniques; accelerating the evaluation of novel agent drug and radiotherapy combinations; and more efficient translational research opportunities, leading to cost savings and research efficiency to improve patient outcomes.

Master protocols offer an innovative platform under which multiple clinical questions can be addressed within a single trial. Due to the complexity of radiotherapy trial setup, cost and research efficiency savings may be more apparent than in systemic treatment trials. Use of this research approach may be the change needed to push forward oncological innovation within radiation oncology.

There is increasing interest in using innovative master protocol trial design in cancer trials. Master protocol trials are developed to simultaneously evaluate more than one intervention and/or multiple different subpopulations within the same overall trial protocol, offering the opportunity to expediate treatment development processes. Most recently, the efficiencies of this approach have been recognized and have been employed in trials of COVID-19 agents (1). In cancer trials, master protocols have emerged in medical oncology to provide a more efficient method for late-stage drug development (2,3). However, there are also significant benefits for a master protocol approach to radiotherapy-focused clinical trials, where novel technology or personalization of treatment, exploring radiotherapy dose, novel drug combinations, and molecular subtyping alongside complex setup often hinders development.

Around 40% of cancer patients are cured using radiotherapy, alone or in combination with other treatments (4). In recent years, there have been three significant areas of development in radiotherapy delivery: novel drug-radiotherapy combinations, personalization of radiotherapy, and novel technological devices. Technological developments within radiotherapy delivery to improve patient outcomes include the use of intensity-modulated radiotherapy, image-guided radiotherapy (including Magnetic resonance imaging [MRI]), stereotactic radiotherapy, and proton therapy (5,6). These new techniques offer the opportunity to deliver greater doses of radiotherapy more precisely to the tumor, while avoiding the adjacent normal tissues, and creates the opportunity to deliver more personalized doses. In radiation oncology, the pertinent clinical question is often not whether the new technology is better, but to what extent, in which indications, and whether it reduces toxicity (7). Addressing this question has previously been limited by heterogeneity of treatment over time, patient selection, and radiation delivery and quality within and across trials. In addition, the therapeutic ratio may be increased through using novel drug-radiotherapy combinations to optimize the potential synergistic relationship between the two approaches in order to improve survival outcomes, while minimizing toxicity (8-10). However, despite clear evidence of the benefit of drug-radiotherapy combinations to enhance efficacy, outside of concurrent chemotherapy there is limited routine use of other systemic agents alongside radiotherapy (11,12). Both technological advances and novel drug-radiotherapy combinations may be exploited to improve the personalization of radiotherapy delivery, including risk and/or biomarker-stratified research. These three key areas of recent development in radiotherapy research offer the opportunity to consider the application of a master protocol, thus optimizing an efficient clinical trial design to bring about practice changing outcomes.

Master protocols may be categorized into three groups: basket, umbrella, and platform (Figures 1-3) (13). Basket designs refer to a trial whereby a targeted therapy is evaluated within multiple disease types with a common molecular characterization or similar underlying patient characteristics (Figure 3). Umbrella designs refer to a trial evaluating multiple therapies within a single disease type, incorporating treatment stratification by, for example, molecular characterization (Figure 2). Platform trials, of which multi-arm, multi-stage (MAMS) design trials are a specific type, are designed to offer a potentially flexible protocol in which multiple (more than two) experimental treatments are considered within a single disease type and may include a common control arm (Figure 1). Prespecified rules to allow the opportunity to add or drop treatments throughout the course of the trial may be incorporated. Each of these approaches allow multiple linked, yet separate, research questions to be evaluated.

**Figure 1.**
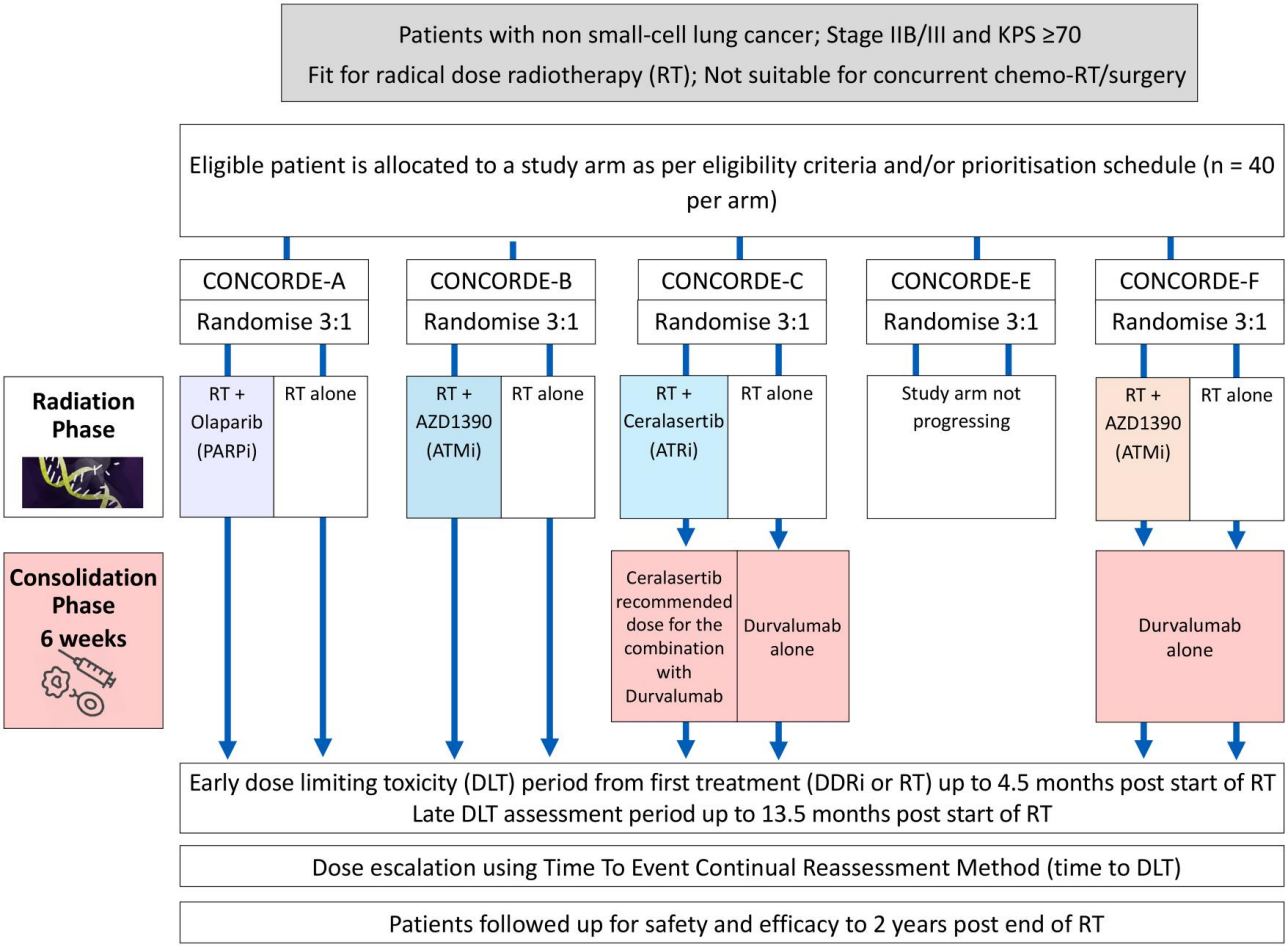
Platform trial—CONCORDE. CONCORDE PARPi = Poly (ADP-ribose) polymerase inhibitors; ATMi = Ataxia telangiectasia mutated kinase inhibitor; ATRi = Ataxia telangiectasia mutated and Rad3-related kinase inhibitor; DDRi = DNA damage response inhibitor.

**Figure 2.**
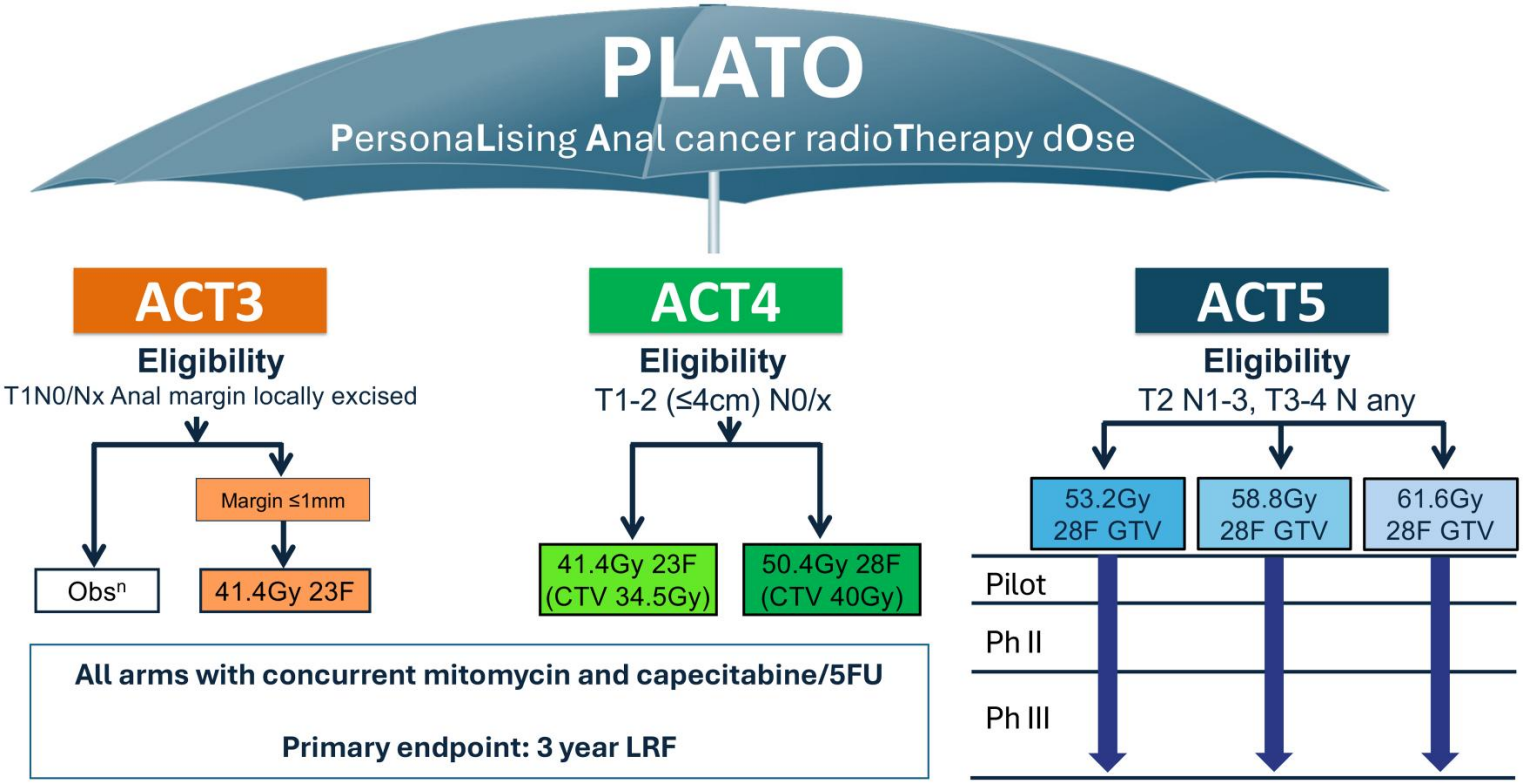
Umbrella trial—PLATO. PLATO = PersonaLising Anal cancer radioTherapy dOse; LRF = local regional failure; GTV = gross tumour volume; F = fraction; 5FU = 5-fluorouracil; Ph = phase.

**Figure 3.**
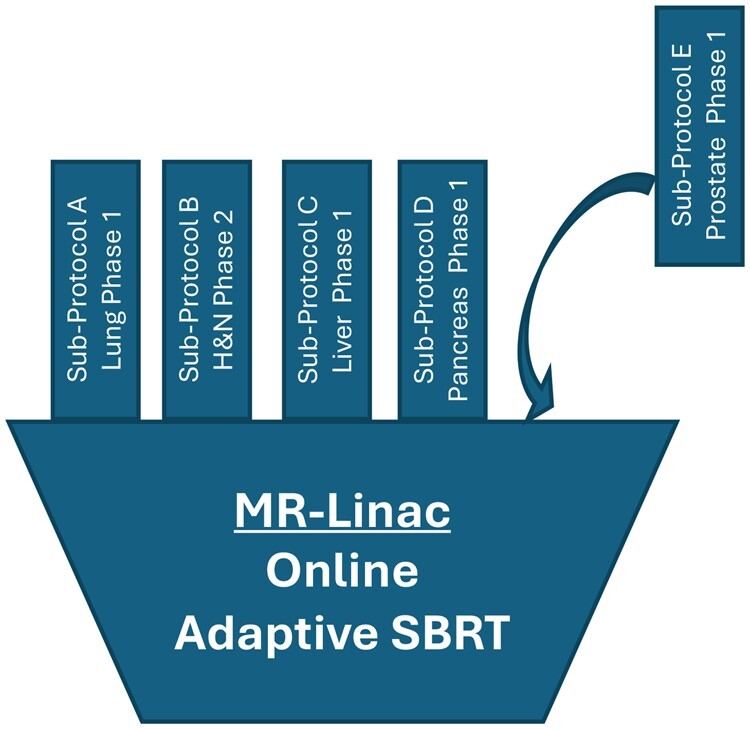
Basket trial—SMART. SMART = Stereotactic Magnetic resonance guided Adaptive Radiation Therapy; SBRT = Stereotactic body radiotherapy.

Although resource requirements to design and develop master protocols can be substantially higher compared to those associated with evaluating a single experimental treatment, efficiencies afforded in recruitment, cost, conduct, and evaluation of multiple therapies or molecularly characterized groups is a clear attraction to researchers. In oncology, there has been a recent surge of literature around methodological considerations and practical implementation of these approaches (2,13-15). A recent landscape analysis identified 83 master protocol trials either conducted or proposed up to July 2019 (16). The majority of these were conducted in the previous five years, and within oncology (91.6%; 76/83). Yet, of the 76 oncology master protocols identified, only three evaluated radiotherapy interventions as a core component of the clinical trial. Similarly, a recent registry review evaluating the uptake of the multi-arm, multi-stage platform approach highlighted only 5% of such trials evaluating radiotherapy or nondrug interventions (17).

Due to the novelty of this approach to design in radiotherapy trials, we sought to evaluate the current use of master protocol approaches for efficient radiotherapy trial design using a comprehensive systematic literature review, including trials currently in setup. Our evaluation showcases the current trial landscape in radiation oncology, summarizing the benefits, limitations, and challenges in this setting by means of example.

## Methods

The Preferred Reporting Items for Systematic Reviews and Meta-Analysis (PRISMA) reporting guidelines were used for this systematic review (18). For the purpose of this review, master protocol trials were defined as a trial that has “one overarching protocol designed to answer multiple questions” (13). Radiotherapy trials were defined as trials in which more than one trial arm included radiotherapy as an intervention (eg, radiotherapy dose modification, novel radiotherapy-drug combinations, radiation devices). To understand the current landscape for use of master protocols in radiotherapy trials, we first reviewed the oncology trials identified by Park et al. (16). Titles were reviewed to identify trials including radiotherapy, and trial characteristics were reviewed to identify trials in which radiation was listed as an intervention. We replicated and updated the MEDLINE, EMBASE, and CENTRAL searches performed by Park et al. to identify additional radiotherapy master protocol publications between July 2019 and July 2022.

Systematic searching of clinical trial registries was performed to identify studies in setup, recruitment, or follow-up that may not have yet been published. The following search strategy was applied to each of the ISRCTN, EudraCT, and ClinicalTrials.gov registries on July 18, 2022: (“platform” OR “umbrella” OR “basket” OR (“master” AND “protocol”)) AND (“radiotherapy” OR “radiation” OR “radiotherapies”). After de-duplication from registries, titles and registry entries were reviewed for each trial identified. Only trials identified as using a master protocol (basket, umbrella, or platform) AND including radiotherapy in more than one trial arm were included for full-text review. Non-English language studies were excluded.

Three reviewers (SB, RS, and AG) independently reviewed all titles, trial characteristics as described in Park et al., abstracts, and trial registry entries identified (16). Full-text publications and available protocols were then retrieved where possible and assessed for eligibility by all reviewers. Data extraction was performed independently to identify key trial design components and to enable individual trial summaries to be produced. Trial interventions were categorized as “drug,” “dose,” and/or “device” to identify the primary focus of the trials’ research questions. An additional category was added to highlight those trials that were also biomarker-driven. The first posted date on clinicaltrials.gov was taken as the trial start date for trials in recruitment. Three trials were selected for detailed summary to describe the benefits and limitations of master protocols in three distinct settings relevant to radiotherapy research: i) radiotherapy-drug combination trials, ii) personalized therapy, and iii) device evaluation. They were selected based on availability of protocol information and the authors’ involvement in the trials, allowing for more detailed insights. We also chose to categorize disease sites according to the aim of the treatment (curative or palliative) to reflect a potentially different focus of the primary research question. A meta-analysis was not performed for this study as this is not intended to represent a systematic review of trial findings. Instead, findings were reviewed using a landscape analysis and were summarized descriptively, reporting on trial characteristics that highlight the benefits, limitations, and challenges of developing and implementing radiotherapy master protocols.

## Results

**Figure 4.**
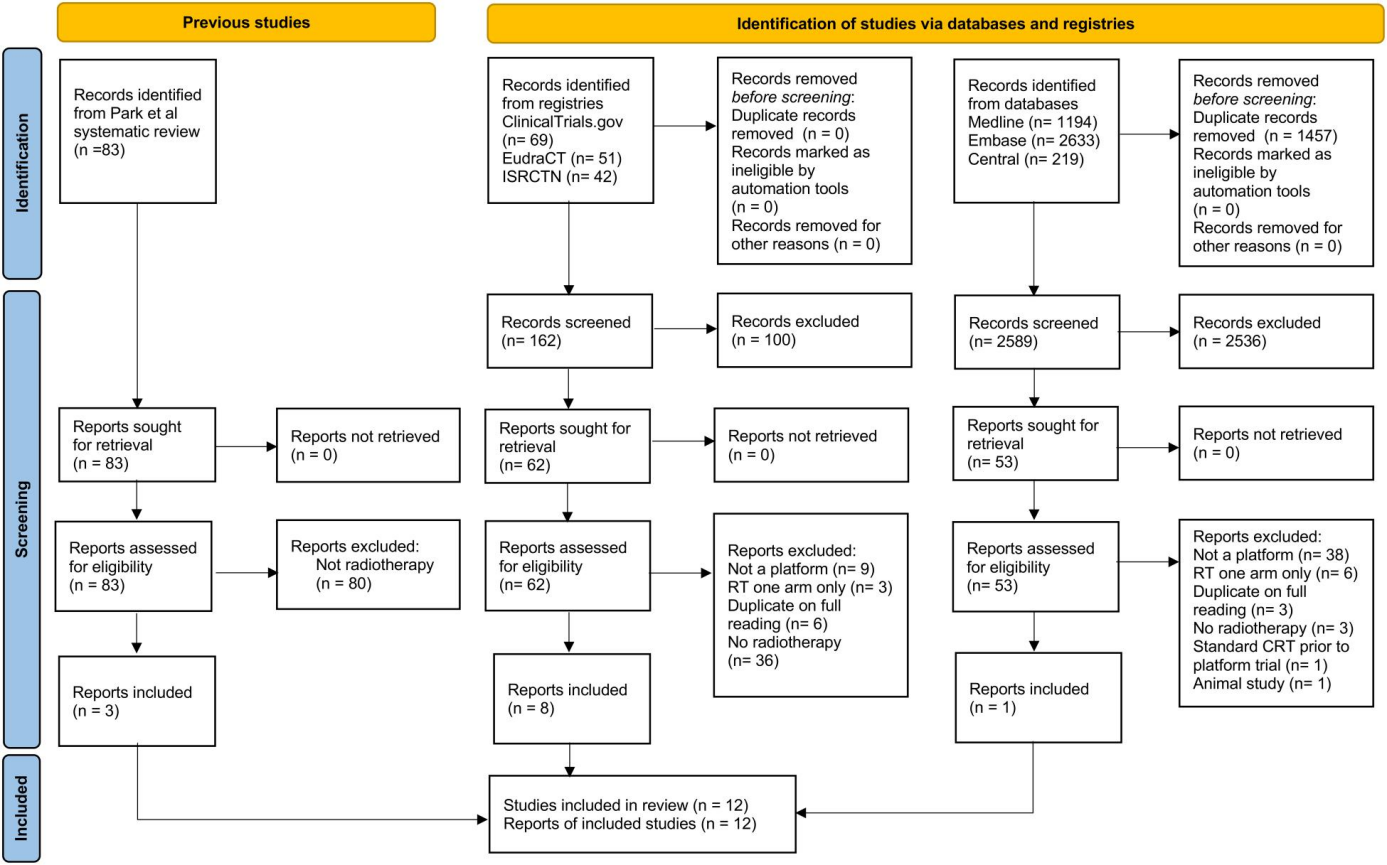
PRISMA Diagram.

Figure 4 summarizes the results of the database and registry searches in the PRISMA flow diagram. Of the 83 oncology master protocol papers identified by Park et al. in their landscape analysis to July 2019 (16), we identified 3 trials with a specific focus on radiotherapy intervention evaluation (Table 1). Upon reviewing clinical trial registries, we identified 51 trials from the European Union Drug Regulating Authorities Clinical Trials (EUDRACT) database, 42 via ISRCTN, and 69 via ClinicalTrials.gov. Once duplicates arising from the Park et al. paper were removed, 8 trials remained. An update of Park et al. (16) MEDLINE, EMBASE, and CENTRAL database searches identified an additional 53 publications for abstract and/or full-text review. Once duplicates were removed, one additional trial was found from these databases, resulting in a total of 12 trials (19). Of the 12 trials included in this review, 4 are platform trials, 3 are umbrella trials (including molecular or risk stratification), and 5 are basket trials (Tables 1 and 2). Full-text protocols and/or publications were obtained for 9 trials.

**Table 1.** Trial details

Study	Identified from^d^	Type of master protocol^b^	Geographical location	Phase	Disease area	Intervention type (1)	Intervention type (2)	Number of experimental arms	Randomised	Biomarker selection	Proposed sample size	Recruitment status	Sponsor; Collaborators/source of funding
AGADIR^a^	Park (14)	Basket	France	II	Advanced solid tumours	Immunotherapy-radiotherapy combination	Drug	6	No	No	247	Recruiting	Academic; Industry
CONFIRM	Registry	Basket	USA	I/II	Gastric, breast, mantle cell, larynx, bladder	MRI image guided radiotherapy	Device	5	No	No	70	Recruiting	Academic; Industry
JUMP	Registry	Basket	USA	I/II (Feasibility)	Prostate, liver, head and neck, recurrent adenocarcinoma	MRI simulator in radiotherapy planning	Device	3	No	No	Not stated	Recruiting	Academic; Industry
SMART	Registry	Basket	USA	I/II	Pancreas; lung; renal, metastatic	Stereotactic MRI guided adaptive radiation therapy	Device	3	No	No	1000	Recruiting	Academic; Industry
UMBRELLA-II	Database	Basket	Netherlands	Feasibility	Prostate, rectal, liver, lung, head and neck, bladder, gastric, esophageal oligometastatic disease	MRI image guided radiotherapy with embedded sub studies	Device	7, with potential to expand	No	No	140	Recruiting	Academic; Industry
C4-MOSART^a^	Registry	Platform	USA	I	Advanced solid tumours	Immunotherapy-SBRT combination	Drug	2	No	No	60	Completed	Academic; Industry
CONCORDE	Registry	Platform	UK	I	NSCLC	DDRi-radiotherapy combination	Drug	Up to 5	Yes	No	200	Recruiting	Academic; Charity and Industry
NCT02764593 (23)	Registry	Platform	USA	I	Head and neck	Immunotherapy-CRT combination	Drug	4	No	No	39	Completed	Academic; Industry
NCT02921256	Registry	Platform	USA, Puerto Rico	II	Rectal	Immunotherapy-CRT combination	Drug	2	Yes	No	348	Completed	Academic; Industry
NCT Neuro Master Match N²M² (NOA-20)	Park	Umbrella	Germany	I/IIa	Glioblastoma	Drug-radiotherapy combination	Drug	7	Yes ^c^	Yes	450	Recruiting	Academic; Charity and Industry
NCT04605562^a^	Registry	Umbrella	China	II	Nasopharyngeal	Immunotherapy-CRT combination	Drug	3	No	Yes	206	Not yet recruiting	Academic; No funding details available
PLATO	Park	Umbrella	UK	II and III	Anal	Radiotherapy dose	Dose personalisation	5	Yes^c^	No; risk stratified	711	Completed	Academic; Charity

a Full protocol not available. NSCLC = non-small cell lung cancer in key.

b Determined by protocol description, or by reviewers if not defined in protocol, following Park et al.

c Randomized in some groups.

d Allocated to 1) Park 2) Registry 3) Database.

**Table 2.** Summary characteristics

	N = 12
**Full protocol/protocol paper identified**	
Yes	9
No	3
**Type of master protocol** ^a^	
Basket	5
Umbrella	4
Platform	4
**Geographical location** ^a^	
China	1
France	1
Germany	1
Netherlands	1
Puerto Rico	1
United Kingdom	2
United States	6
**Disease setting** ^a^	
Curative	10
Palliative	4
**Phase**	
I	3
I/II	4
II	3
II and III	1
Feasibility	1
**Recruitment status**	
Not yet recruiting	1
Recruiting	8
Active, not recruiting	2
Completed	1
**Focus of research question** ^a^	
Radiotherapy-drug combination	7
Dose personalization	1
Device evaluation	4
Novel-agent radiotherapy combination: biomarker driven	2

a Not mutually exclusive.

The earliest trial start date was recorded from October 2016 (NCT02921256) and ranged to 1 trial currently in setup (NCT04605562). As of November 2023, 4 trials have stopped recruitment and 1 of these has reported final results (20), 8 are currently recruiting, and 1 is not yet recruiting. Half of the trials are led from the United States (NCT02921256 with Puerto Rico), 1 from China (NCT0460562) (30), and the rest from Europe. There was a spread across the development pathway, highlighting radiotherapy master protocols are being used across each of phase I, II, and III. One trial stated it was a feasibility study without stating phase (19). Most trials were supported by both academic sponsors and industry funding, with 3 European trials also supported by charity funding.

The 4 platform trials cover several different solid tumors: 3 covering head and neck cancer (one including oropharyngeal cancer), rectal, anal, and non-small-cell lung cancer. They all evaluate the role of novel agent drug combinations with (chemo)radiotherapy. Three are phase I trials, and 1 is a phase II in locally advanced rectal cancer, with the platform designed to allow expansion of the novel agent-radiotherapy combinations. Three are evaluating the addition of immunotherapy to standard-of-care (chemo)radiotherapy. The 5 basket trials are evaluating a novel treatment process. Four are evaluating the role of devices, in this case the use of MRI in delivery of radiotherapy, and one involves a novel approach to combining an intratumoral agent and radiotherapy. Two studies involve the use of MRI for image guidance in a metastatic setting; SMART evaluates the use of adaptive MR-guided treatments in delivery of stereotactic radiotherapy (SBRT) (22) and CONFIRM in delivery of fractionated image guided radiotherapy (28). UMBRELLA-II is a feasibility basket trial with embedded sub-studies (described as a basket-umbrella trial), evaluating MRI-guided radiotherapy in multiple cancer sites (both metastatic and curative) and includes sub-studies to optimize MR-Linac workflow and MR-sequence protocols efficiently (19). JUMP (25) evaluates the use of an MRI simulator to plan radiotherapy (and includes both metastatic and curative patients), and AGADIR (29) evaluates the role of atezolizumab, intra-tumoral G100, and radiotherapy (to injected lesion and SBRT). Two of the 3 umbrella trials include true molecular biomarker stratification within their trial design with allocation into the experimental arms based on gene profiling within its trial design [National Clinical Trial (NCT) Neuro Master Match N^2^M^2^ (31)] and nasopharyngeal carcinoma [NCT04605562 (30)]. The third umbrella trial includes risk stratification by tumor stage [PLATO (28)], with research questions focusing on dose personalization. Experimental arms included treatment escalation for high-risk groups and de-escalation for low and intermediate risk groups. Eight trials had no stratification (due to a lack of current predictive biomarkers); however, 5 included a planned translational component within the trial protocol. Statistical designs used included time to event continuous reassessment method and Bayesian continuous toxicity monitoring in phase I; feasibility and estimation in phase I/II; A'Hern single stage, Simon’s two-stage and a multi-stage binomial design in noncomparative phase II trials, and use of a single control arm for comparison with concurrently randomized experimental arms in a randomized phase II trial [NCT02921256 (21)]. Only 1 study (PLATO) used different statistical designs across the umbrella, including a randomized phase II with no formal comparison to control and a randomized comparative phase III design with pilot and phase II interim analysis. No formal statistical design was noted for 3 trials, from the information available (19,24,30).

The following representative trials were selected to describe the benefits and limitations of master protocols in 3 distinct settings, as described: i) Box 1: radiotherapy-drug combinations: CONCORDE (27); ii) Box 2: personalized therapy: PLATO (28); and iii) Box 3: device evaluation: SMART (22).

Box 1. Representative radiotherapy-drug combination trial—CONCORDE (Figure 1)

CONCORDE is a phase I platform trial evaluating multiple DNA damage response inhibitors (DDRis) in combination with radiotherapy (RT) in patients with advanced non-small-cell lung cancer not fit for concurrent chemoradiotherapy (27). The academic-charity-industry partnership offers a unique opportunity to evaluate a suite of agents with similar mechanisms of action via an efficient platform approach. The time to event continuous reassessment method (TiTE-CRM) is applied to each of up to 5 DDRi arms (32), standardizing methodology and parameter estimation across the trial. The platform design enables patients to be recruited to alternative experimental arms, similar in style to a flip-flop approach (33), ensuring ongoing recruitment. Inclusion of a concurrent calibration arm (receiving radical radiotherapy alone) enables benchmarking of dose limiting toxicities, a concept not commonly observed in phase I dose-finding drug-only trials. The control and experimental arms were recently updated for the later arms to include maintenance immunotherapy based on changing clinical practice, demonstrating the flexibility of the platform approach. Concurrent calibration data on up to 50 patients across the platform enables exploration of molecular characterization and associations with outcomes. CONCORDE is a discrete platform including up to only 5 experimental arms, ensuring a clear study-end and outputs to inform later-stage development.

There are inevitably challenges associated with a platform approach in this setting of both phase I and radiotherapy-drug combinations. As with all master protocols, the development alongside multiple subprotocols can extend protocol timelines. In the case of phase I industry partnerships, contracting timelines can impact setup. The phase I nature of the drugs means changes often need to be made to dosing schedules or dose levels for exploration. Statistically, the design needs to be updated and evaluated to ensure acceptable operating characteristics. In the platform setting, this often needs addressing across a number of arms, in parallel.

The relevance of master protocols for dose finding in the era of targeted therapies is discussed by Polley and Cheung (34). The additional benefits specifically in the radiotherapy-drug combination setting include standardization of radiotherapy technique and quality assurance, inclusion of control patients to aid interpretation of attribution, and the ability to recruit across multiple arms, while patients are followed up for long dose limiting toxicity (DLT) observation periods.

**Patient perspective:** CONCORDE has formed a patient and public involvement (PPI) group of 6 representatives, who play various trial roles, including overseeing safety review, inputting into day-to-day decision-making, and reviewing patient information sheets (PIS) and protocol updates. Patient co-researchers have been instrumental in the study’s development, and their input remains crucial throughout the trial, in particular the review of new patient documents such as diaries and PIS. Patients are approached for entry into a single study arm within the platform to avoid the complexity of assessing multiple PIS. If eligible for multiple study arms, a prespecified prioritization schedule determines which arm they are approached for. Randomization occurs within the study arm, and patients are randomly assigned to either the DDRi+RT combination or RT only after consent.

Box 2. **Representative personalized therapy trial—PLATO** (Figure 2)

Squamous cell carcinoma of the anus is rare, with approximately 10 000 patients diagnosed in the United States each year (35). Chemoradiotherapy (CRT) is the established standard of care. The PLATO (PersonaLising rAdioTherapy dOse in anal cancer) umbrella trial is designed to address the question of radiotherapy dose modification in 3 anal cancer trials (ACT3, 4, and 5) across the locoregional disease spectrum (36). Clinically, the PLATO trial offers patient recruitment across the disease spectrum in a rare cancer, where trial setup and feasibility of recruitment may hinder practice changing outcomes. The use of an umbrella approach to address questions about radiotherapy dose modification based on TNM stage risk levels is applied in the absence of a biomarker target. ACT3 and ACT4 question selective use of radiotherapy or the use of dose reduction, respectively, in patients with more favorable characteristics to minimize toxicity while not impacting oncological outcomes. ACT5 dose escalation aims to improve local regional control while not significantly increasing toxicity. It is important to note the timely nature of dose modification trials. With routine use of precision radiotherapy techniques, such as volumetric modulated arc therapy (VMAT), it is now possible to effectively “dose paint” radiotherapy fields, meaning that areas of high and lower dose can be included heterogeneously within a single treatment for a patient. The ability to sculpt dose and avoid normal tissue structures means that it is possible to increase the dose of radiotherapy delivered in a way that was not possible before due to high rates of severe toxicity. The ability to apply different statistical designs to each ACT3, 4, and 5 is another benefit of the umbrella approach. Notably, where data collection and follow-up schedules differ between groups, the design of a standard database is limited.

The opportunities for translational research also increase with inclusion of patients across different tumor stages, as questions incorporating biomarker-driven questions stratified by tumor stage are possible. For example, research questions regarding p16 positive status as a predictive biomarker for better response to radiotherapy and, therefore, an indicator in addition to tumor size for dose de-escalation may be better answered if patients with high and lower risk disease receiving multiple different radiotherapy doses are included in the analysis. Predictive models of toxicity may also be analyzed more effectively when a dose spectrum to normal tissues is available.

Finally, a calculation from the use of a single master protocol rather than 3 separate trials estimated a cost savings of around 20% (£450 000) in trial and site unit costs at the time of grant application in 2016. The cost savings calculated were based on significantly lower staff costs due to reduced trial administration burden and efficiencies in contracts and site setup. However, further cost savings may also have occurred through faster recruitment and broader opportunities for translational research in a rare cancer.

**Patient perspective:** Patients were invited to 1 of the 3 trials based on eligibility criteria (TNM stage); therefore, multiple different interventions were not discussed. However, if patients became ineligible for 1 of the trials (eg, due to progression on their planning scan), the option to take part in one of the other trials, if eligible, could be offered. This demonstrates the potential efficiency of an umbrella design whereby patients still have a trial option available; thus, a positive action can arise from an unfortunate clinical scenario.

Box 3. **Representative device evaluation trial—SMART** (Figure 3)

The MR-Linac is cutting-edge technology that has only recently been developed and was US Food and Drug Adminiatration (FDA) approved in 2017. The rationale for the increased clinical efficacy of this device compared to conventional X-ray-guided linear accelerators is based on its ability to improve image guidance during radiation therapy. This includes providing images with superior soft tissue contrast and enabling real-time imaging and adjustment of targeting during treatment (“online adaptive radiation therapy”), all of which could help to decrease the amount of healthy tissue exposed to radiation and ensure that the tumor is being optimally treated with radiation. The use of a phase I and II master protocol trial of stereotactic magnetic resonance imaging–guided adaptive radiotherapy (37) is the first master protocol trial to test a radiation oncology device.

In radiation oncology, the pertinent clinical question is often not whether the new technology is better, but to what extent, in which indications, and whether it reduces toxicity, in order to establish how improvements in technology translate into patient benefit. Addressing this question has previously been limited by heterogeneity of treatment over time, patient selection, and radiation delivery and quality within and across trials.

In contrast to drugs, many radiation oncology technologies are adopted in the absence of prospective, level I evidence. Differences exist in clinical implementation and standards of evaluation of radiation devices when compared to oncology drugs based on different regulatory approval pathways. For the FDA, for example, radiation devices are classified as medium-risk devices and are approved via the premarket notification (510[k]) pathway based on a finding of “substantial equivalence” to predicate devices and require only preclinical supporting data. Although classified as medium risk, radiation devices have the capacity to inflict substantial harm in the event of an error leading to undertreatment or toxicity. There is a need for more thorough clinical evaluation of new radiation oncology technologies to demonstrate the value of radiation treatments and to minimize patient harm.

For the evaluation of a new device, the use of a basket master protocol trial provides an efficient means to develop a single, cancer-agnostic protocol encompassing multiple cancer types with the same design, statistical considerations, logistics, and infrastructure. The flexible protocol allows new disease sites to be added as subprotocol amendments. The technical and practical considerations of implementing a device within a patient pathway in radiation oncology is a substantial challenge and should be considered a complex intervention. The general eligibility, treatment techniques, quality assurance, and clinical assessment are specified by the master protocol, and more specific eligibility criteria, treatment specifications, and disease-specific assessment are detailed separately for each sub-study. The protocols have the potential to be multi-institutional, hastening accrual and maximizing generalizability.

A critical difference between trials of drug therapies and devices is the importance of operator competency and aptitude with the device. In the past, inadequate user training and quality assurance has compromised outcomes and confounded interpretation, limiting our ability to directly compare technologies. Through the efficient use of the master protocol process, the master protocol document may address the two key concerns, quality assurance and user competency, when implementing a new device. The document may serve as both a teaching document and include prospective quality assurance to standardize techniques and ensure high-skill implementation of the new device. Incorporating training and quality assurance procedures into the master protocol minimizes the resources needed per sub-study compared with traditional trial design and maximizes standardization. This is particularly valuable for studies of rare indications for which intensive credentialing programs may be most important but not economically realistic with a traditional study design. Umbrella training and credentialing also simplifies the addition of new sub-studies across multiple study sites, further adding to the efficiency of completing these trials.

Furthermore, the ability to pool select endpoints across different cancer types that share similar anatomical locations, and thus radiation technique and toxicity considerations, can allow more rapid assessment of feasibility and safety of treating tumors with these properties.

**Patient perspective:** Patients understood they were receiving standard-of-care doses of radiation but delivered on a new radiation machine and understood the technique was being tested in a variety of different cancer types. Feedback from patients was positive; they were grateful to have had the opportunity to be treated with new technology. By establishing safe delivery of standard doses, future research will look to innovate and adjust radiation dose and delivery in future studies.

## Discussion

To our knowledge, this is the first landscape analysis of master protocol trials of radiotherapy. To capture the use of this novel trial methodology, as well a systematic review of published trials, the review of the literature was expanded to include protocol publications and multiple international clinical trial registries with the aim of including all master protocol trials proposed or conducted to date. Lack of consistent Medical Subject Headings (MeSH) and nomenclature across trial registries hampered duplication detection at screening and may have resulted in missing trials. For example, the one trial found from the updated database search does have a clinicaltrials.gov record but was not found in any registry searches. The majority of the 12 master protocol trials involve the investigation of novel agents in combination with radiotherapy, supported by academic-industry collaborations. This reflects the findings of a previous landscape analysis of all trials, which found most master protocol trials were evaluating novel agents in adult oncology patients (16).

In contrast to the review by Park et al., only one radiotherapy master protocol trial has published its final results; therefore, reflection on the true efficacy of this approach is broadly theoretical (20). However, the efficiencies in shared radiotherapy protocols and quality assurance processes across multiple trial arms are evident at time of trial setup and will impact cost (7). In addition, one of the key differences between the master protocol trials designed for medical oncology in comparison to radiation therapy is the lack of biomarker-driven trials. Park et al. (14) identified 18 within oncology; we identified only 2. This lack of biomarker-driven trials in radiotherapy has already been identified as a research area in urgent need of focus and a working group developed to address this (9,10). However, as highlighted in the Park et al. review, use of other baseline patient characteristics (eg, staging as used in PLATO) can also be used to drive the focus of personalization of interventions in the absence of biomarker targets. In comparison to the spread across the different types of master protocol trials within our review, Park et al. found almost half of the 83 master protocols identified were a basket design (47%; n = 39). Although not explicitly reported, a review of the supplementary information provided by Park et al. reveals a greater proportion of trials in patients with metastatic disease rather than in a curative setting, as expected within systemic therapy trials, which likely accounts for this difference in choice of trial design.

The use of master protocol trials in radiotherapy has the potential to offer real value in three key research areas identified in this review: i) accelerating the evaluation of the (biomarker-driven) novel agent drug and radiotherapy combinations to enhance the efficacy of radiotherapy response, often in a curative setting (38); ii) evaluating radiotherapy dose modification across the spectrum of a single disease site, enabling additional translational research opportunities within both tumor and normal tissue response modeling; iii) sharing the efficiencies of a single radiotherapy protocol to evaluate novel radiotherapy technology (devices and new approaches to treatment delivery such as protons, MR-Linac, SBRT), offering efficiencies in quality assurance and establishing user competency.

The value of a master protocol outlining radiotherapy procedures is of significant value over and above the research opportunities. The workflow outlining technical procedures for simulation and immobilization, image guidance, target and normal structure delineation, daily setup, radiotherapy planning optimization, and treatment delivery can be standardized (if relevant) across trial arms and the subprotocols defining disease or treatment specific characteristics then provided. A shared protocol significantly reduces duplication of work in areas where radiotherapy processes are disease or treatment-site agnostic.

Although not specific to radiotherapy trial design, the statistical considerations surrounding the use of the master protocols are somewhat specific to the trial underdevelopment. General considerations as well as issues surrounding the use of nonconcurrent control arms, adding, and dropping arms and multiplicity assumptions are discussed throughout the literature (15,39-41). Master protocols offer flexibility to apply different statistical designs to each arm or each component within the trial, addressing different research questions and potentially differing phases of trial development, as seen within the PLATO trial. Complex novel designs can be used within master protocols, as, for example, in CONCORDE (32) and UMBRELLA-II (19), without additional complexities compared to multiple single trials. Where the same statistical design is applied across multiple arms, efficiencies with regard to numbers of patients compared to multiple traditional two-arm trials have been observed (42). Similarly, within phase I trials, where radiotherapy techniques may change and historical data are unreliable, the use of a pooled control across arms in a platform approach can be particularly beneficial to enable benchmarking and interpretation, resulting in more meaningful data compared to multiple single-arm trials and offering an additional valuable data source for translational research, particularly in rare populations. Radiotherapy research offers a unique, data-rich source including pre- and post-treatment imaging as standard for radiomics evaluation, and the ability to model tumor response on radiotherapy imaging. Combining multiple arms within a master protocol offers the opportunity to evaluate complex research questions through rich, multimodal data assets and the potential to provide digital comparator data for future clinical trials.

Our review highlights the general lack of master protocol trials in radiotherapy being conducted. This may be due to lack of awareness or lack of examples specifically within radiotherapy. Master protocols are increasingly being adopted across academic clinical trials units as a more efficient approach to clinical trial delivery than multiple traditional two-arm clinical trials, and general guidance to support efficient development and conduct is paramount. A recent publication from experienced UK trials units provides practical guidance on the development and delivery of these trials in a bid to increase uptake and to support the wider use of these approaches (2). Practical considerations when developing a trial proposal including consideration of how to approach patients, protocol structure, criteria for and implementation of new arms, and trial oversight committee structures have been highlighted (43), as well as the need to be mindful of the working environment and the impact on individual training and progression needs where studies are inevitably large-scale and complex (44). Supplementary Table 1 (available online) summarizes the benefits and challenges of master protocol trials in radiotherapy.

Regarding limitations, one of the key challenges with evaluating a novel methodological area of research is the potential variability in terminology, nomenclature, and indexing of the different terms used to described master protocol trials within the databases and by the researchers developing these trials. However, building on an existing published systematic review search strategy, and supplementing this with rigorous review of three international clinical trial registries, we aimed to encapsulate this novel research approach. Second, the review included English language only publications; however, this effect was minimized as no trial has yet published results.

Future areas to work on translational efficiency within master protocol trial design may include the use of real-world data linkage to create contemporaneous controls or digital comparators and inclusion of pre- and post-treatment imaging for radiomics evaluation and modeling of tumor and normal tissue response.

The drive toward more efficient clinical trials is ever increasing, often with a need to answer multiple clinical questions within a single trial. Master protocols offer an innovative platform under which many of these questions can be addressed, as they provide one overarching protocol designed to answer multiple research questions with a broad set of objectives, offering the opportunity to expedite treatment development processes. By maximizing efficiencies in radiotherapy protocol development and quality assurance, as well as financial and often statistical benefits, this research framework minimizes the resources needed to deliver innovative trials to push forward oncological innovation within radiation oncology.

## Supplementary Material

djae084_Supplementary_Data

## Data Availability

Data sharing requests will be considered on written request through contacting a.gilbert@leeds.ac.uk.
