# Supplementary material for: The use of master protocols for efficient trial design to evaluate radiotherapy interventions: a systematic review
Source: J Natl Cancer Inst. 2024 Apr 27;116(8):1220–9. doi: 10.1093/jnci/djae084 (PMC11308198; doi:10.1093/jnci/djae084)
Supplement: djae084_Supplementary_Data [file djae084_supplementary_data.pdf]

Supplementary Table

Supplementary Table 1: Summary of benefits and challenges of Master Protocol trials in Radiotherapy

BENEFITS	CHALLENGES
**Radiotherapy specific**	**Radiotherapy specific**
Standardised radiotherapy techniques and quality assurance	May require longer period of learning and development than single trial
Shared control arm - reduces overall sample size and avoids use of historical control arm (ensures contemporary RT techniques)	-
Efficiencies testing novel agent-RT combinations particularly with longer DLT observation periods required	Multiple contracts with different industry collaborators
Wider opportunities for translational research, including biomarker, imaging, toxicity modelling	-
**General**	**General**
Significant cost savings, particularly on staff costs, contracts and site set up	Longer and more complex set up of the master protocol than a single trial
Shared governance structures e.g. committees	May require more frequent commitment from governance teams
Shared patient co-researcher involvement: potential to support each other	More complex trial design for patient co-researcher involvement; may require additional training
Efficiencies on documentation e.g. protocol, CRFs, reports, ethics	Longer development processes for documentation than single trial
-	Adaptations including adding arms requires significant protocol amendments throughout the trial
Recruitment may be more efficient (multiple options available)	Design is more complicated to understand which may deter patients
Efficiencies at analysis stage (e.g. common analysis plans possible, similar trial endpoints and data structures)	-
